# Fatal Carney Complex in Siblings Due to *De Novo* Large Gene Deletion

**DOI:** 10.1210/jc.2017-01045

**Published:** 2017-07-26

**Authors:** Maria Stelmachowska-Banaś, Wojciech Zgliczyński, Piotr Tutka, J. Aidan Carney, Márta Korbonits

**Affiliations:** 1 Department of Endocrinology, Centre of Postgraduate Medical Education, Warsaw 01-809, Poland; 2 Department of Experimental and Clinical Pharmacology, University of Rzeszow, Rzeszow 35-310, Poland; 3 Department of Laboratory Medicine and Pathology, Mayo Clinic, Rochester, Minnesota 55905; 4 Department of Endocrinology, William Harvey Research Institute, Barts and the London School of Medicine, Queen Mary University of London, London EC1M 6BQ, United Kingdom

## Abstract

**Context:**

Carney complex (CNC) is a rare multiple neoplasia syndrome involving cardiac, endocrine, neural, and cutaneous tumors and a variety of pigmented skin lesions. CNC can be inherited as an autosomal dominant trait, but in about one-third of patients, the disease is caused by *de novo* mutation in the *PRKAR1A* gene localized on chromosome 17q22-24. Most of the mutations include single base substitutions and small deletions/insertions not exceeding 15 base pairs. Recently, large germline *PRKAR1A* deletions have been described and may cause a more severe phenotype.

**Case Description:**

Herein, we report the cases of two siblings with CNC with a *de novo* large deletion of 107 kb at 17q24.2 associated with acromegaly in both and primary pigmented nodular adrenocortical disease, cardiac myxoma, and lethal metastatic melanotic schwannian tumor at the age of 27 years in one of them, supporting the hypothesis that large deletions of *PRKAR1A* lead to severe disease.

**Conclusions:**

To our knowledge, this is the first description of familial CNC in siblings in which neither parent carried the deletion in blood-derived DNA, suggesting that one of them had germ cell mosaicism for this deletion. Testing for large gene deletions should be obtained in all patients who meet the diagnostic criteria for CNC but do not have a *PRKAR1A* mutation by Sanger sequencing.

## Teaser

We studied Carney complex in siblings due to parental germ cell mosaicism of large *PRKAR1A* deletion, causing lethal metastatic melanotic schwannian tumor in the proband.

**Figure 1.**
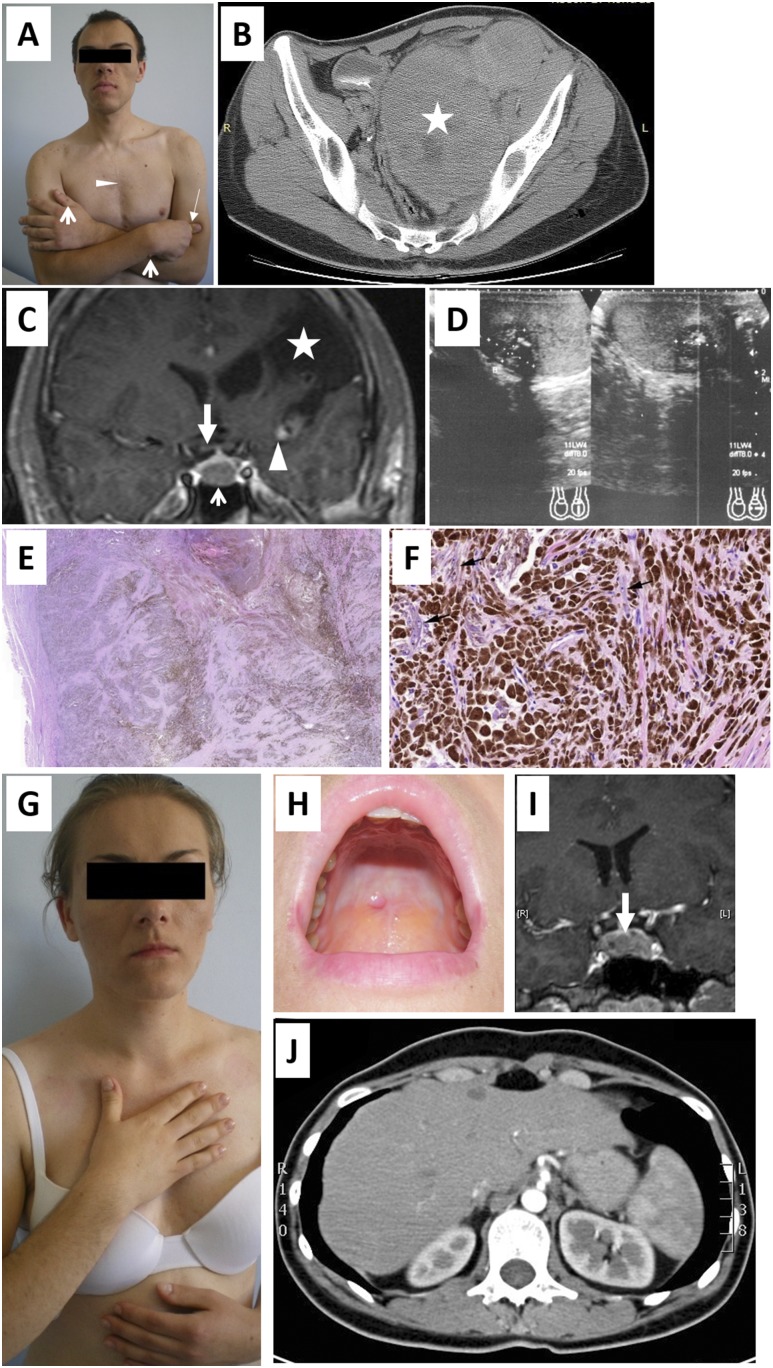
(A) Acromegalic features in the proband. Note chest scar from previous cardiac myxoma operation (triangle), typical facial features (wide nose, prognathism, prominent eyebrow and thickened lips), cutaneous myxomas on left thumb and right wrist (open arrows), large left hand, and spastic right hand due to the hemiplegia (closed arrow). He also had enlarged feet and tongue. (B) Computed tomography of the pelvis of the proband revealed a 14.0-cm × 8.5-cm tumor (star). (C) Gadolinium-enhanced coronal magnetic resonance imaging scan of the head of the proband showed four abnormalities: a pituitary adenoma (open arrow), a large left hemispheric cavity in the left cerebral hemisphere after childhood embolic stroke (star), multiple middle cerebral artery aneurysms (triangle) resulting from myxoma emboli stroke and the probable cause of the patient’s epileptic fits, and right optic nerve atrophy (closed arrow). Image is slightly fuzzy due to patient movement artifact. (D) Scrotal ultrasound of the proband revealed bilateral calcified testicular tumors. (E) Metastatic melanotic schwannian tumor. Low-power magnification image showed sheets of pigmented tumor cells with degenerative fibrosis surrounded by a thick fibrous capsule (hematoxylin and eosin, ×40). (F) High-power magnification revealed pigmented polygonal and spindle cells with a few nonpigmented elongated tumor cells (black arrows) (hematoxylin and eosin, ×200). (G) Mild acromegalic features in the sister. (H) Myxoma on the hard palate in the sister. (I) Gadolinium-enhanced magnetic resonance imaging scan revealed an intrasellar pituitary microadenoma in the sister. (J) Representative image of the computed tomography scan showing normal adrenal glands in the sister, despite the abnormal biochemistry results.

A 26-year-old man presented with typical features of acromegaly (Fig. 1A). At the age of 8 years, he developed right faciobrachial paralysis and right-sided blindness due to middle cerebral and central retinal artery embolism leading to right optic nerve atrophy. A 4-cm left atrial mass (myxoma) was removed surgically. At age 15 years, he had an epileptic seizure. Magnetic resonance imaging revealed multiple middle cerebral artery aneurysms. A right external ear myxoma was removed at age 16 years. At 24 years, he presented with a 4.7-cm × 4.3-cm × 6.5-cm left popliteal mass composed of spindle and epithelioid cells with cytoplasmic melanin staining positive for melanoma markers, HMB45, and melan-A. It was interpreted as metastatic melanoma, although no skin melanoma was found. 18F-fluorodeoxyglucose positron emission tomography revealed a 14.0-cm × 8.5-cm pelvic tumor (Fig. 1B) that was unresponsive to chemotherapy, including bleomycin, vincristine, lomustine, dacarbazine, cisplatin, vinblastin, and dacarbazine, followed by ipilimumab 1 year later. The patient was referred to the endocrine department due to fatigue and low blood pressure; ipilimumab-induced hypophysitis was suspected. Physical examination revealed right upper limb hemiparesis, divergent right strabismus, and typical acromegalic features (Fig. 1A). His height was 175 cm (midparental height 165.5 cm). There were pigmented eyelid spots, left thumb and right wrist myxomas, goiter, and palpable bilateral testicular nodules. High insulinlike growth factor 1 (IGF-1) and nonsuppressed growth hormone (GH) was found on glucose loading (Supplemental Table 1). Low morning adrenocorticotropic hormone, cortisol, and dehydroepiandrosterone sulfate levels indicated secondary adrenal insufficiency; hydrocortisone replacement was initiated. Elevated follicle-stimulating hormone and luteinizing hormone and decreased total testosterone level suggested primary hypogonadism. Prolactin level was within normal limits. Magnetic resonance imaging revealed a pituitary macroadenoma (Fig. 1C). Ultrasound examination of neck revealed a nodular goiter (thyroid volume 32 mL). Scrotal ultrasonography showed bilateral testicular tumors with calcifications (Fig. 1D). Echocardiography results were normal, suggesting that there was no recurrence of his previous cardiac myxoma. Long-acting somatostatin analogue and palliative pelvic radiotherapy (2000 cGy) was administered. Pathological reevaluation of the popliteal tumor revealed an invasive spindle and epithelioid cell melanin-producing tumor with necrosis, prominent nucleoli, and occasional mitoses. The tumor lacked features of psammomatous melanotic schwannoma, laminated calcified bodies, fat, and a peripheral bony shell. The lesion was reinterpreted as a malignant melanotic schwannian tumor (Fig. 1E and 1F). The clinical course and pathological findings were consistent with Carney complex (CNC). DNA sequencing of exons 2 to 11 of the *PRKAR1A* gene revealed no mutation. The patient died of progression of the malignant schwannoma at age 27 years.

**Figure 2.**
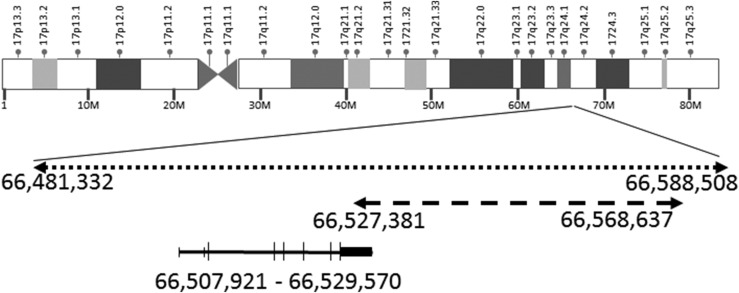
Representative depiction of chromosome 17 with the area marked of the maximum size of the deletion (dotted arrow, from base-pairs 66,481,332 to 66,588,508; numbers are according to the GRCh37/hg19 human genome assembly) and the minimum size of deletion (dashed arrow, from 66,527,381 to 66,568,637). *PRKAR1A* gene (located at 66,507,921 to 66,529,570) is represented with coding exons (longer lines) and untranslated exons (shorter lines/area).

The proband’s 25-year-old sister (165 cm tall, midparental height 152.5 cm) noticed enlargement of hands, feet, nose, lower jaw, and tongue over the past 5 years (Fig. 1G). On examination, she had a small goiter, skin and mucosal pigmented spots, mild hirsutism, acne, small lesions on the nipple, and hard palate (myxomas) (Fig. 1H). Hormonal analysis revealed elevated IGF-1, nonsuppressed GH, and mildly elevated prolactin level (Supplemental Table 1). Pituitary magnetic resonance imaging revealed a 5.3-mm microadenoma (Fig. 1I). Following long-acting somatostatin analogue treatment, she underwent transsphenoidal surgery (GH- and prolactin-positive adenoma, Ki-67 <1%) with normalization of GH, prolactin and IGF-1. Echocardiography showed no tumors. Transvaginal and neck ultrasound respectively showed polycystic ovaries and nodular goiter (thyroid volume 22 mL). Repeated 24-hour urinary free cortisol (UFC) measurements were normal, but a 1-mg overnight dexamethasone test did not suppress cortisol, and there was a “paradoxical” increase in UFC on high-dose dexamethasone administration (Supplemental Table 1). Computed tomography scan of the adrenals was normal (Fig. 1J). As DNA sequencing of *PRKAR1A* of her brother’s DNA was negative for *PRKAR1A* mutation, a comparative genomic hybridization array was performed on her and her asymptomatic parents. She had a 17q24.2 [minimum region 41 kb (66,527,381 to 66,568,637), maximum region, including the *PRKAR1A* gene, 107 kb (66,481,332 to 66,588,508)] deletion (Fig. 2), whereas neither of the parents carried the deletion. Paternity was confirmed. Written informed consent was gained from patients and family members.

## Discussion

CNC can occur in families or sporadically as a result of a *de novo* mutation (one-third of cases) in *PRKAR1A*. Most mutations include single base substitutions or small (<15 base pair) deletions/insertions (1, 2). Large deletions (20%) are associated with a more severe phenotype (3) and should be searched for in patients who meet diagnostic criteria for CNC (Supplemental Table 2) but do not have a *PRKAR1A* mutation by Sanger sequencing. In “CNC plus,” a 2.3-Mb deletion of 17q24.2-q24.3 covering the *PRKAR1A* gene has been associated with posterior laryngeal cleft, growth retardation, microcephaly, moderate intellectual disability, and numerous freckles/lentigines (3, 4). Other syndromes, such as Dubowitz syndrome, may also be associated with deletions on chromosome band 17q24.2 and a strong phenotypic resemblance (5).

CNC has an almost 100% penetrance, and ~70% of patients with CNC have an affected parent. In this family, most likely one of the parents had gonadal mosaicism for this deletion.

The proband died of progression of a malignant melanotic schwannian tumor, initially interpreted as metastatic malignant melanoma of unknown origin. Melanotic schwannoma can be mistaken for melanoma (6). Finding a deep paraspinal pigmented spindle cell tumor and no cutaneous melanoma should prompt consideration of melanotic schwannoma (7). CNC can be associated with young-onset melanotic schwannoma (2) with and without psammoma bodies (8). Pathological evaluation is a poor predictor of behavior of melanotic schwannoma, even in patients with histologically benign-appearing tumors. Clinical follow-up, available in 26 cases, showed local recurrences in 35% of patients and metastases in 42%. Recently, a new nomenclature has suggested renaming melanotic schwannomas as “malignant melanotic schwannian tumors” (9). Loss of PRKAR1A protein may have value in distinguishing melanotic schwannoma from metastatic melanoma; classical melanomas have shown retained protein expression of PRKAR1A, whereas 7 of 21 melanotic schwannomas had loss of PRKAR1A (9).

Cardiac myxomas are the most severe complication of CNC and responsible for the high mortality of the patients with CNC. Early detection and regular screening of CNC patients with echocardiography are essential (2). Our proband had an embolic stroke at age 8 years complicated by later epilepsy due to multiple aneurysms of the middle cerebral artery, resulting from myxoma emboli. The association of cardiac myxoma and multiple intracranial aneurysms has rarely been documented. The aneurysms result from establishment of cardiac myxoma emboli in the vasa vasorum of the peripheral arteries, the growth of which damages the internal elastic lamina and weakens the vessel wall. The weakened wall then dilates, resulting in typical angiographic findings: peripherally located fusiform aneurysms (10). Although sporadic cardiac myxomas (75% left, 20% right atria) usually develop in middle-aged women, in CNC, the mean age of onset is 20 years; they are often multiple, occur in any or all chambers, affect the sexes equally, and commonly recur at the initial resection site (2). About 7% of cardiac myxomas are associated with CNC.

The most common endocrine tumor in CNC is primary pigmented nodular adrenocortical disease (PPNAD) resulting in adrenocorticotropic hormone–independent hypercortisolism (Supplemental Table 2). Histological evidence of PPNAD has been found in almost every individual with CNC who underwent autopsy, but a number of patients have had asymptomatic PPNAD. Although the sister had a typical adrenal presentation (no hypercortisolemia, normal adrenal computed tomography, lack of dexamethasone suppression, and a “paradoxical” UFC increase on the Liddle test), the proband had secondary hypocortisolism probably due to ipilimumab-induced hypophysitis. In PPNAD, cortisol production can be variable and sometimes cyclical, resulting in a paradoxical cortisol rise following dexamethasone administration. PPNAD continues to be a radiological diagnostic challenge because of the small size of the pigmented nodules and the small size of the adrenal glands (2).

Up to 75% of patients with CNC exhibit asymptomatic elevation of GH, IGF-1, or prolactin, and 10% to 12% have acromegaly with detectable pituitary adenoma usually presenting at or after the third decade.

Our proband had primary hypogonadism, likely due to bilateral large-cell calcifying Sertoli cell tumors. However, the role of recent chemotherapy cannot be ruled out.

## Summary of Learning Points

• All young patients with myxomas should be assessed for possible CNC.
• Testing for large gene deletions should be obtained in all patients who meet the diagnostic criteria for CNC but do not have a *PRKAR1A* mutation by Sanger sequencing.
• Differential diagnosis of melanoma and melanotic schwannoma can be challenging.
• When blood-derived DNA does not show a CNC mutation in parents with more than one offspring with CNC, gonadal mosaicism could be the explanation.
